# Human Immune System Increases Breast Cancer-Induced Osteoblastic Bone Growth in a Humanized Mouse Model without Affecting Normal Bone

**DOI:** 10.1155/2019/4260987

**Published:** 2019-05-09

**Authors:** Tiina E. Kähkönen, Mari I. Suominen, Jenni H. E. Mäki-Jouppila, Jussi M. Halleen, Azusa Tanaka, Michael Seiler, Jenni Bernoulli

**Affiliations:** ^1^Pharmatest Services, Turku 20520, Finland; ^2^Taconic Biosciences, Rensselaer, 12144 NY, USA

## Abstract

Bone metastases are prevalent in many common cancers such as breast, prostate, and lung cancers, and novel therapies for treating bone metastases are needed. Human immune system-engrafted models are used in immuno-oncology (IO) studies for subcutaneous cancer cell or patient-derived xenograft implantations that mimic primary tumor growth. Novel efficacy models for IO compounds on bone metastases need to be established. The study was performed using CIEA NOG (NOG) mice engrafted with human CD34+ hematopoietic stem cells (huNOG) and age-matched immunodeficient NOG mice. Bone phenotyping was performed to evaluate baseline differences. BT-474 human breast cancer cells were inoculated into the tibia bone marrow, and cancer-induced bone changes were monitored by X-ray imaging. Bone content and volume were analyzed by dual X-ray absorptiometry and microcomputed tomography. Tumor-infiltrating lymphocytes (TILs) and the expression of immune checkpoint markers were analyzed by immunohistochemistry. Bone phenotyping showed no differences in bone architecture or volume of the healthy bones in huNOG and NOG mice, but the bone marrow fat was absent in huNOG mice. Fibrotic areas were observed in the bone marrow of some huNOG mice. BT-474 tumors induced osteoblastic bone growth. Bone lesions appeared earlier and were larger, and bone mineral density was higher in huNOG mice. huNOG mice had a high number of human CD3-, CD4-, and CD8-positive T cells and CD20-positive B cells in immune-related organs. A low number of TILs and PD-1-positive cells and low PD-L1 expression were observed in the BT-474 tumors at the endpoint. This study reports characterization of the first breast cancer bone growth model in huNOG mice. BT-474 tumors represent a “cold” tumor with a low number of TILs. This model can be used for evaluating the efficacy of combination treatments of IO therapies with immune-stimulatory compounds or therapeutic approaches on bone metastatic breast cancer.

## 1. Introduction

In many of the most common cancers including breast, prostate, and lung cancers, the majority of metastases are formed at the skeleton [1–4]. These bone metastases are incurable and remarkably decrease the quality of life at end-stage disease [1–6]. Currently, the treatment of bone metastases is based on conventional chemotherapeutics and compounds that inhibit bone resorption such as bisphosphonates, the RANK-ligand antibody denosumab, and bone-seeking radium-223 dichloride, but they only decrease tumor growth and prolong the time of induction of skeletal-related events (SREs) in patients [7]. To overcome the incurable bone metastases, many therapies are under investigation and the most promising approaches come from the field of immuno-oncology (IO) [6, 8]. When cancer cells migrate to the bone, they can stay quiescent for decades before the clinically detectable bone metastasis is observed, and the immune system is hypothesized to have a role in this process [5, 8, 9]. Bone marrow has a unique microenvironment in many aspects, also with regard to the immune milieu [4, 9]. The bone marrow contains many immune cells, including mostly B cells, immunosuppressive cells such as myeloid-derived suppressor cells (MDSCs), and regulatory T cells (Tregs) [4, 9]. Tumors have many means to avoid elimination by immune cells [8, 10, 11]. Tumor and/or immune cells can become immune evasive by expressing immune-regulatory molecules such as the programmed cell death protein 1 (PD-1), programmed death-ligand 1 (PD-L1), or cytotoxic T lymphocyte-associated protein 4 (CTLA-4) [8, 10, 11]. As the bone is a site of many immune-suppressive cells, it is rational to think that this microenvironment would support the immune-evasive phenotype even further. Furthermore, the bone microenvironment is a reservoir of many growth factors that from the early events of tumor cell dissemination support cancer cell survival and tumor growth in the bone [5, 6, 9, 12].

In bone metastasis, the bone and immune system are linked also through the immune cells and bone-resorbing osteoclasts differentiating from the same CD34+ hematopoietic stem cells (HSCs) [13–15]. Besides having the same origin, T and B cells have a role in the maintenance of bone homeostasis, which has created a basis for the osteoimmunology concept [15]. Osteoimmunology is a complex field that has just recently gained the interest of a larger audience. The main findings can be divided into three categories: [1] immune cells and inflammatory cytokines have catabolic effects in the bone, [2] immune cells have anabolic effects in the bone, and [3] the bone marrow partly regulates the development of immune cells [15]. Tumor necrosis factor (TNF) is one important cytokine that not only mediates the inflammatory processes but also increases bone resorption by inhibiting the differentiation of bone-forming osteoblasts and promoting differentiation of bone-resorbing osteoclasts [4, 15]. In addition, T cells, such as Th1 cells, and Th17 cells especially, can promote osteoclast differentiation by inducing expression of the receptor activator of the nuclear factor kappa-B ligand (RANKL) mediated by interleukin- (IL-) 17 or interferon gamma (IFN*γ*) [15]. T and B cells express RANKL which then increases osteoclast differentiation and bone resorption [4, 15]. Contrary to catabolic effects, immunosuppressive cells such as Tregs can inhibit osteoclastogenesis mediated by IL-4 and transforming growth factor beta (TGF*β*) [9, 15]. Other effects of immune cells on bone cells, for example, on osteocytes, are under investigation.

About 80% of potential drug candidates fail in the translation of preclinical findings to clinical efficacy [12, 16, 17]. To improve this, more predictive animal models should be used in preclinical testing. Ideally, these models in immuno-oncology would combine the human tumor and human immune cells in the correct metastatic microenvironment. The most commonly used efficacy models in oncology are subcutaneous tumor models in mice. These models lack tumor-stroma interactions in the correct tissue microenvironment, which are important in cancer initiation, growth, and progression [12, 18]. More sophisticated models include orthotopic and metastasis models, and metastasis models can be divided into systemic and local models [3, 12, 18]. In local metastasis models, tumor cells are directly inoculated to the metastatic site. The advantage of this approach includes direct tumor formation on-site, easier detection, and more homogeneous tumor growth, thus reducing the number of mice used in a study.

Suitable models in immuno-oncology were for a long time limited to using syngeneic models [16, 19, 20]. These models are fast and effective, but in many cases, they fail to mimic the human conditions, and mouse cancer cells are, for example, differentially dependent on certain cytokines regulating immune cell responses [16, 20]. If preclinical findings are only relying on syngeneic models, there is a risk of misleading findings that do not translate to clinical efficacy [16, 19]. A humanized mouse is a human-mouse chimera transplanted with human cells, tissues, or organs [16, 21]. In this article, when referring to humanized mice, we discuss the human immune system- (HIS-) engrafted models. These humanized models are created on the basis of super-immunodeficient mice, such as NOG or NSG, to avoid the development of graft-versus-host disease [16]. These models were developed on the nonobese diabetic (NOD) inbred mouse strain and have the homozygous null mutation in Prkdc scid and a targeted null (NSG) or the functionally null (NOG) mutation in the gamma chain of the IL-2 receptor (IL-2R*γ*) leading to attenuation of mouse T, B, and NK cell development [16, 21, 22]. When NOG mice are engrafted with human CD34+ (hCD34+) HSCs, they differentiate into functional human immune cells [16, 21, 23]. hCD34+ HSC-engrafted mice have proven to be effective in studies when evaluating immunological effects on tumors [16, 24]. The engraftment with hCD34+ HSCs is the best option for long-term maintenance of the hematopoietic system in mice [16, 20]. The advantage of using humanized mice is the species-specific interactions between human tumor cells and human immune cells [16], which further enables efficacy testing of fully humanized antibodies.

To study bone metastasis, novel platforms such as humanized bone organ models [25, 26], 3D culture models [27], or models of the human bone in mice [28] have been established. As stated in several publications, more models for immuno-oncology concentrating on bone metastasis are needed [9, 12, 19]. The aim of this study was to establish the first breast cancer bone growth model in humanized mice. This novel model would then combine the human tumor, bone, and human immune system and could be used for preclinical validation of new IO therapies and therapeutic combinations.

## 2. Materials and Methods

### 2.1. Animals and Animal License

Human immune system-engrafted mice (huNOG; HSCCB-NOG-F, Taconic Biosciences) were used in the study. Briefly, the humanized mice were produced by causing a mild myeloablation to super-immunodeficient CIEA NOG® (NOG) mice (NOD.Cg-Prkdc^scid^II2rg^tm1Sug^/JicTac, Taconic Biosciences) with low-dose irradiation and engrafting them with human cord blood-derived CD34+ HSCs at 3-5 weeks of age. Nonirradiated age-matched NOG mice were used as controls. The number of mice in each group was 6. The animal experiments were carried out with an approval from the National Animal Experimentation Board of Finland. The mice were sacrificed by inhalation of CO_2_, the death was confirmed by cervical dislocation, and the tissue samples were collected for analysis.

### 2.2. Cell Culture

BT-474 human breast cancer cells were purchased from the American Type Culture Collection, authenticated and tested to be negative for common mouse pathogens and for mycoplasma. The cells were cultured in DMEM/F-12 (Sigma-Aldrich) supplemented with 10% heat-inactivated fetal bovine serum (iFBS, Sigma-Aldrich) in a humidified incubator at +37°C and 5% CO_2_. 1 × 10^6^ of BT-474 cells suspended in 20 *μ*l of 1x phosphate-buffered saline (PBS) was used per inoculation to the mice. Before and after the inoculation, cell viability was determined (NucleoCounter®, NC-200, ChemoMetec) and it was above 80%.

### 2.3. Intratibial Model

The method of intratibial injection has been previously described [29]. Briefly, prior to the inoculations, the mice received an analgesic (Temgesic; buprenorphine, 1 mg/kg s.c.; Indivior) at least 30 minutes before the inoculations. The mice were anesthetized with isoflurane (Attane Vet, Isoflurane, Piramal Healthcare), and the cells were inoculated into the bone marrow of the right proximal tibia. After the inoculations, pain management was done by administration of Temgesic (0.2 mg/ml) to the drinking water for two consecutive days.

### 2.4. Serum Markers

Blood samples were collected from the *vena saphena* after animal warming for 5 min under a heating lamp. 200 *μ*l of blood was collected into tubes including the clotting activator (Microvette 200 Z-Gel, Sarstedt Ag & Co.). The blood samples were collected before the inoculation of the cancer cells (at study day -3) and before sacrifice (at study day 56). The blood samples were processed into serum as instructed by the manufacturer. The serum samples were analyzed for TRACP5b (tartrate-resistant acid phosphatase 5b, MouseTRAP and BoneTRAP Assays), PINP (procollagen type I N-terminal propeptide, Rat/Mouse PINP EIA), and CTX-I (C-terminal telopeptide of type I collagen, RatLaps EIA, all from IDS Systems). The measurements were done according to the protocol provided by the manufacturer, and the plates were read with the VICTOR2™ Multilabel Counter (PerkinElmer).

### 2.5. X-Ray Imaging

Cancer-induced bone changes (i.e., bone lesions) were monitored by X-ray imaging at 4, 6, and 8 weeks after inoculation of the cancer cells. The X-ray images were taken with an UltraFocus DXA (Faxitron Bioptics LLC) with automatic energy and exposure time. The bone lesion area in the tumor-bearing tibia was quantified with MetaMorph (Molecular Devices LLC) image analysis software.

### 2.6. DXA

For bone mineral density and content analysis, dual X-ray absorptiometry (DXA) was used. The mice were imaged with an UltraFocus DXA with automatic energy and exposure time. The software automatically defined the amount of calcified tissue (bone map). The bone map was used for analysis of bone mineral density (BMD, mg/cm^2^) and bone mineral content (BMC, g) from a predefined area which was 6 mm long and started below the growth plate. This analysis was performed both from tumor-bearing and intact (healthy) tibia. To analyze cancer-induced changes in the bone, BMC and BMD values from the intact tibia were subtracted from the values obtained from the tumor-bearing tibia of the same mouse. This allowed to quantitate the cancer-induced increase in BMD and BMC in each mouse separately.

### 2.7. *μ*CT

The tibiae were fixed in 10% neutral buffered formalin (NBF, FF Chemicals) for at least 72 h and stored in 70% ethanol. After fixation, the tibiae were imaged with microcomputed tomography (*μ*CT; SKYSCAN 1078, Bruker) using the settings of 50 kV, 195 *μ*A, 5.3 *μ*m as the pixel size, and 0.45° step size. The images were analyzed for cortical and trabecular bone parameters separately. The measurement region started below the most proximal site of the uncalcified cartilage of the epiphyseal growth plate, and the measured area was 4 mm long. The analysis was performed for the tumor-bearing and healthy tibia separately using the same settings.

### 2.8. Histology and Histomorphometry

The tibia samples were decalcified in EDTA (BDH Chemicals) and processed into paraffin blocks (Tek III Paraffin Wax, Sakura, Netherlands). Midsagittal 4 *μ*m FFPE sections were obtained from each sample. The sections were stained with hematoxylin and eosin and Orange G (HE-Orange G, reagents from Sigma-Aldrich and Acros Organics) for basic histological evaluation of the tumor and bone and with pararosaniline (Sigma-Aldrich) for the staining of osteoclasts using standard methods. The stained sections were scanned with a digital slide scanner (Pannoramic Scanner 250, 3DHISTECH) and analyzed with the Pannoramic Viewer and HistoQuant (3DHISTECH). Tumor and bone areas were defined in each section from the growth plate to 5 mm distance to the distal tibia and analyzed by color-thresholding. Also, the number of osteoblasts and osteoclasts was analyzed from these images.

### 2.9. Immunohistochemistry

Immunohistochemical stainings were performed using a Lab Vision Autostainer (Thermo Fisher Scientific). Shortly, 4 *μ*m FFPE tissue sections were deparaffined in xylene and hydrated in a decreasing ethanol series. Antigen retrieval was performed in a pretreatment module (Lab Vision) using heat-induced epitope retrieval in Tris-EDTA (pH 9) at +98°C for 20 min. The following primary antibodies were used: CD45 (common leukocyte marker, 2B11+PD7/26/16), CD3 (T cell, BSR10), CD4 (T helper cell, BSR4), CD8 (cytotoxic T cell, BSR5), CD20 (B cell, BSR6), PD-1 (programmed cell death protein 1, BSR1), PD-L1 (programmed death-ligand 1, ZR3, all from Nordic BioSite), and CTLA-4 (cytotoxic T lymphocyte-associated protein 4, BSB-88, BioSB). ER (estrogen receptor, SP1), PR (progesterone receptor, SP2), and HER2 (human epidermal growth factor receptor 2, SP3; all from Spring Bio) were stained as tumor markers. All primary antibodies were incubated for 30 minutes in RT. Endogenous peroxidase activity was blocked with 3% H_2_O_2_ and polymer-based HRP detection (Nordic BioSite), and high-contrast DAB was used for detection and visualization. Human multitissue sections were used for positive and negative controls. Representative images with indicated magnifications are presented.

### 2.10. Statistical Analyses

The statistical analyses were performed with R software (http://www.r-project.com), and the figures were produced by GraphPad Prism 7 software. The statistical tests used varied between the measurements, and the different statistical tests are described in the figure legends. Statistical significance is marked as NS = nonsignificant, ^∗^ *p* < 0.05, ^∗∗^ *p* < 0.01, and ^∗∗∗^ *p* < 0.001 in the figures.

## 3. Results and Discussion

### 3.1. Immune Cells in the Humanized Mice

**Figure 1:**
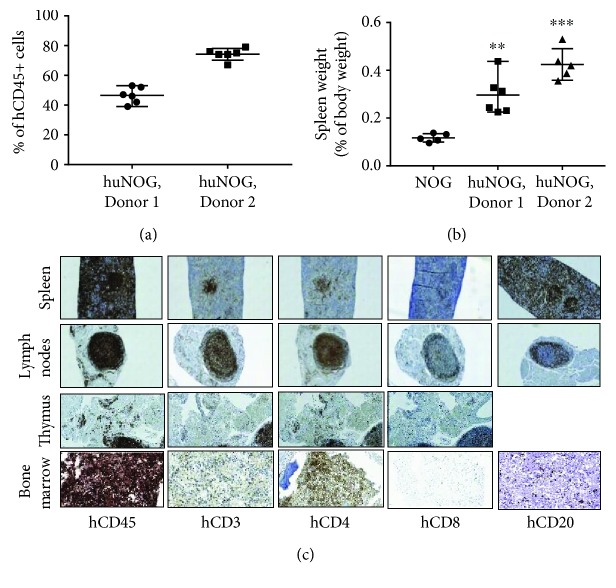
Immune cells in huNOG mice. (a) Chimeric ratio of hCD45/mCD45+ cells (%, median ± min/max) in huNOG mice engrafted with cells from two different donors (Donor 1 and Donor 2). (b) Spleen weight relative to body weight at sacrifice (%, median ± min/max). Prior to statistical analysis, the data was transformed using logarithmic transform. Statistical analysis was performed using ANOVA and pairwise comparisons using Dunnett's test. Statistical significance is marked as ^∗∗^ *p* < 0.01 and ^∗∗∗^ *p* < 0.001. (c) Representative images of immunohistochemical stainings for different human immune cells (hCD45: common leukocyte antigen, hCD3: T cells, hCD4: T helper cells, hCD8: cytotoxic T cells, and hCD20: B cells) in immune-related organs of huNOG mice. Magnification 10x for the spleen, lymph nodes, and thymus and 40x for the bone marrow.

The production and characterization of humanized mice has been previously described [16, 22, 23]. By 17 weeks postengraftment, the CD34+ HSCs have differentiated to mature human immune cells. This was detected by measuring the chimeric ratio of human/mouse CD45+ cells (Figure 1(a)). The chimeric ratio between the mice engrafted with the cells obtained from two different donors was overall high, and it was 50% for Donor 1 and 80% for Donor 2 (Figure 1(a)). The increased quantity of human immune cells was associated with increased spleen weight (Figure 1(b)). In the human blood, mostly myeloid cells are observed and T cells are the second most common immune cell type [20]. From the total number of T cells observed in humans, 45-75% are circulating in the blood, and these cells consist of 25-60% of CD4+ and 5-30% of CD8+ cells [9]. In the blood of humanized NOG, NSG, or SRG mice, B cells are the most common, followed by T cells and myeloid cells [20]. Even though the humanized mouse models recapitulate the distribution of human immune cell populations, they still have proportional changes in the immune cell quantities compared to humans [20].

Human immune cells in immune-related organs of huNOG mice were analyzed by immunohistochemistry (Figure 1(c)). A high quantity of CD45+ cells was observed in the spleen, lymph nodes, thymus, and bone marrow (Figure 1(c)). Higher quantities of CD3+ T cells, mostly comprising of CD4+ T helper cells, were observed in the spleen and lymph nodes compared to the thymus (Figure 1(c)). CD8+ cytotoxic T cells were observed mainly in the lymph nodes (Figure 1(c)) and CD20+ B cells in the spleen and lymph nodes (Figure 1(c)). The number of immune cells in these organs varied to some extent between the mice, but nevertheless, all human immune cells were present in each individual mouse. The human immune cells were detected as larger cells in the bone marrow (Supplement 1). When these cells were stained with CD45, a high-intensity staining was obtained (Figure 1(c)). Otherwise, the intensity of the staining varied between the sections and individual mice. Overall, some CD3-, CD4-, and CD8-positive T cells were observed in the bone marrow together with CD20+ B cells (Figure 1(c)). Immune cell distribution is similar in the human and mouse bone marrow [20]. B cells, myeloid cells, and T cells are observed in the bone marrow of immunocompetent mice [20], and this correlates with our finding of immune cells in the bone marrow of humanized mice.

When these findings are put to the bone metastasis context, the bone marrow has a limited number of CD8+ cytotoxic T cells that would be able to kill the tumor cells. The role of CD8+ cells in regulation of tumor growth in the bone has been shown to be crucial [9]. Simultaneously, the bone marrow contains a large number of immunosuppressive cells that further enhance the tumor growth locally [9].

### 3.2. Bone Phenotype in Humanized Mice

CD34+ cells are the progenitors for immune cells but importantly, in the context of the bone, also progenitors of bone-resorbing osteoclasts [13–15]. Because these cells have the same origin, it would be rational to think that they would be linked also to the regulation and function of each other. In fact, bone cells and especially bone-forming osteoblasts are necessary for HSC maintenance [15]. Before engraftment of HSCs, a low-dose irradiation is applied to the mice to cause a mild myeloablation that improves the engraftment of HSCs. In this study, one of our main questions was how does this affect the bones of huNOG mice, and more specifically, are there any differences in the bones at baseline due to irradiation or differences caused by the immune cells?

**Figure 2:**
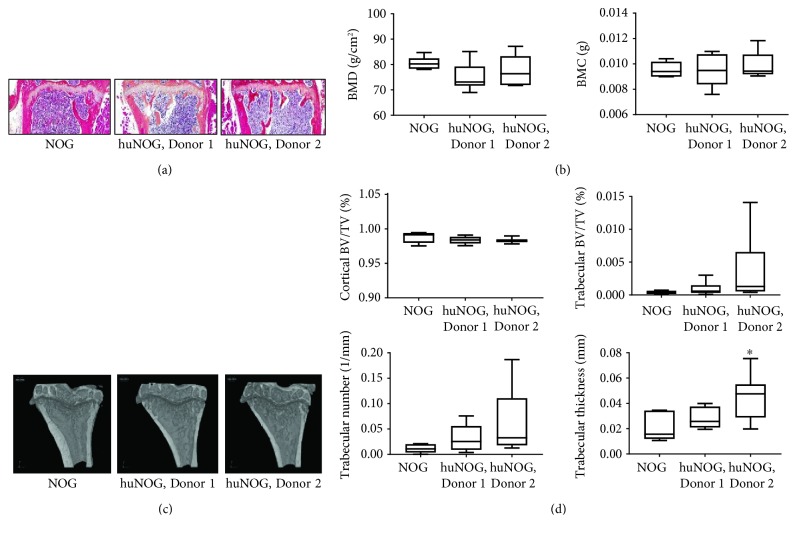
Bone phenotype in NOG and huNOG mice. (a) Representative HE-stained sections of the healthy tibia, magnification 10x. (b) BMD (g/cm^2^, mean ± IQR25%±min/max) and BMC (g, mean ± IQR25%±min/max) in the healthy tibia. Statistical analysis was performed using ANOVA. No statistical differences were observed (*p* > 0.05). (c) Representative 3D reconstructions of the healthy tibia. (d) Cortical bone volume per tissue volume (BV/TV; %, mean ± IQR25%±min/max), trabecular BV/TV (%, mean ± IQR25%±min/max), and trabecular number (1/mm, mean ± IQR25%±min/max). Statistical analysis was performed using ANOVA and multiple comparisons using Dunnett's test. No statistical differences were observed (*p* > 0.05). Trabecular thickness (mm, mean ± IQR25%±min/max). Statistical analysis was performed using ANOVA and pairwise comparisons using Dunnett's test. Statistical significance is marked as ^∗^ *p* < 0.05.

**Figure 3:**
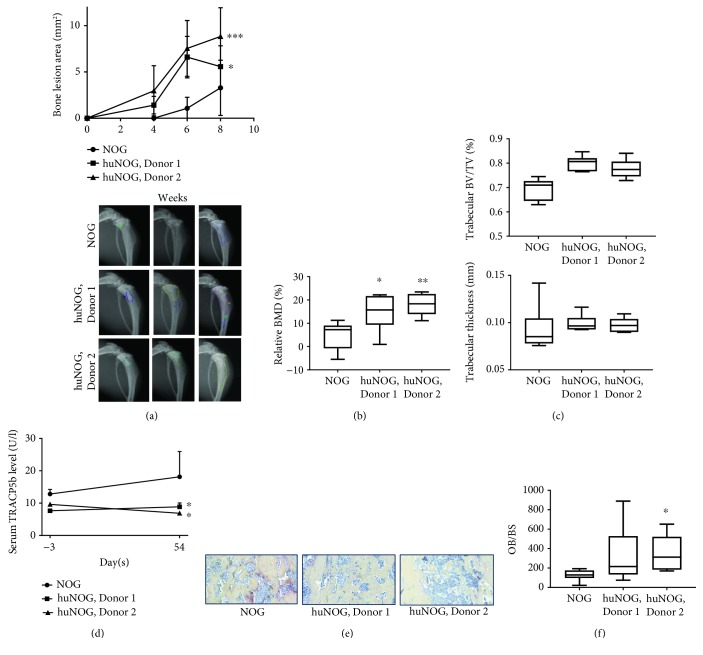
Tumor-induced bone changes in NOG and huNOG mice. (a) The bone lesion area at 4, 6, and 8 weeks after cancer cell inoculation (mm^2^, mean ± SEM). The data was modeled using a linear mixed-effect model and comparisons by model contrasts. The obtained *p* values were adjusted for multiple comparisons. Statistical significances are marked as ^∗^ *p* < 0.05 and ^∗∗∗^ *p* < 0.001. (b) BMD in the tumor-bearing tibia relative to BMD in the healthy tibia (g/cm^2^, mean ± IQR25%±min/max). Statistical analysis was performed using ANOVA and pairwise comparisons using Dunnett's test. Statistical significance is marked as ^∗^ *p* < 0.05 and ^∗∗∗^ *p* < 0.001. (c) Trabecular bone volume per tissue volume (BV/TV; %, mean ± IQR25%±min/max) and trabecular thickness (mm, mean ± IQR25%±min/max). Prior to statistical analysis, the data was transformed using logarithmic transform. Statistical analysis was performed using ANOVA. No statistical differences were observed (*p* > 0.05). (d) Mouse TRACP5b serum levels (U/l, mean ± SEM). The data was modeled using a linear fixed-effect model, and the comparisons were carried out using model contrasts. The obtained *p* values were adjusted for multiple comparisons. Statistical significance is marked as ^∗^ *p* < 0.05. (e) Representative TRACP stainings from histological sections, magnification 20x. (f) Quantitation of the osteoblast number on the bone surface (OB/BS, mean ± IQR25%±min/max). Statistical analysis was performed using ANOVA and pairwise comparisons using Dunnett's test.

Our results showed no significant changes in the bone structure compared to immunodeficient NOG mice based on HE staining of the intact tibias (Figure 2(a)). 2/12 huNOG mice had fibrotic areas in the bone marrow (Supplement 1). Interestingly, the bone marrow fat was completely absent in huNOG mice whereas in NOG mice the bone marrow fat was observed (Figure 2(a)). In the normal bone marrow, adipocytes are usually found with HSCs in the proximal tibia and femur and the amount is usually stabilized at about 12 weeks or later, depending on the mouse strain [30, 31]. Generally, in a bone metastasis model, the marrow fat can have a dual effect: [1] an effect on bone cells/bone mass and [2] direct effects on tumor cells. Bone marrow adipocytes can regulate the bone mass by decreasing the activity of alkaline phosphatase (ALP) and the expression of transcription factor RUNX2, which is important for osteoblast differentiation [30]. However, adipocytes can also induce osteoclast differentiation and activity [30, 31]. Based on these facts, bone marrow adipocytes regulate the balance between bone resorption and formation. The second point addressed is the effects of adipocytes on tumor growth. Adipocytes store and secrete various metabolic factors, growth factors, and cytokines [32–34], and they can promote tumor growth locally in the bone [33, 35]. Our results showed that huNOG mice had no bone marrow adipocytes and the tumors grew better compared to those of the NOG mice (Figure 3(a)). This is controversial to the observations by others as stated above, and it can be concluded that bone marrow adipocytes are not essential for tumor growth in this model. Additionally, bone marrow adipocytes can reduce the number of HSCs *in vitro* [31]. According to our findings, the lack of the bone marrow fat was associated with increased hematopoiesis and leukocyte differentiation as shown by others [31]. It is possible that the lack of bone marrow adipocytes enabled the good engraftment of HSCs in the mice.

No changes in BMD or BMC were observed in the intact tibia of huNOG and NOG mice (Figure 2(b)). To identify possible changes in the bone structure, *μ*CT imaging and analysis of the tibia were performed and representative images are shown in Figure 2(c). No changes were observed in the cortical bone volume (Figure 2(d)). Also, no significant changes but a tendency towards increased trabecular bone volume were observed in huNOG mice (Figure 2(d)), which seemed to be in accordance with the quantity of human immune cells in these mice (Figure 1(a)). When looking into more details in the trabecular bone changes, a trend towards increased trabecular number and a significant increase in trabecular thickness were observed in the Donor 2 mice (Figure 2(d)). The number of osteoclasts was decreased in huNOG mice compared to NOG mice as evaluated by quantitation of the osteoclast number from histological sections of the tibia (Supplement 2), which is also supported by the measurement of TRACP5b serum levels from the mice (Figure 3(d)). There were no differences in the number of osteoblasts (Supplement 2). Additionally, the bone formation marker PINP and the resorption marker CTX-I were measured to study differences in bone turnover. PINP levels were lower in Donor 2 mice, while CTX levels were lower in Donor 1 mice compared to NOG mice (Supplement 2). The PINP/CTX ratio that indicates bone turnover ratio was lower in Donor 2 mice compared to NOG mice (Supplement 2).

To explore if the CD34+ HSCs also differentiate to human osteoclasts in humanized mice, serum levels of human TRACP5b were analyzed. The assay showed some cross-reaction to mouse TRACP. However, human TRACP levels seemed to be elevated in huNOG mice, although the levels were still low and barely detectable (data now shown).

Our findings observed in huNOG mice indicate some changes in bone phenotype but no concerns in using these mice in bone-related studies. The decreased number of osteoclasts may be due to irradiation at a young age. Further studies could provide a deeper understanding of the functional/molecular changes in bone cells and their relation to immune cells in the model. The lack of the bone marrow fat may prevent using these mice in some specific studies. Even though the humanized mice have been widely used, this is the first study evaluating their bone phenotype.

### 3.3. BT-474 Human Breast Cancer Cells Induced Extensive Osteoblastic New Bone Growth in Humanized Mice

BT-474 human breast cancer cells induced osteoblastic new bone growth in the inoculated tibia (Figure 3(a)). Quantifiable bone changes appeared later in NOG mice compared to huNOG mice (Figure 3(a)). When the bone lesion area was quantitated at 4 weeks, no lesions were observed in NOG mice, but in huNOG mice, the lesions were already 1.5 mm^2^ in Donor 1 mice and 3 mm^2^ in Donor 2 mice (Figure 3(a)). Growth of osteoblastic bone lesions was quantified from X-ray images at all time points. The bone lesion area was larger in huNOG mice compared to NOG mice (Figure 3(a)). Also here, some differences between the donors were observed in bone lesion growth but the change between the donors was nonsignificant (Figure 3(a)). A larger bone lesion area was associated with increased BMD in the tumor-bearing tibia in huNOG mice (Figure 3(b)). The increased BMD was associated with a trend towards increased trabecular bone volume and thickness (Figure 3(c)). To study the cause behind increased BMD and the trabecular bone, we analyzed changes in osteoclast number. TRACP5b serum levels were higher in NOG mice already at baseline, and the levels slightly increased towards the end of the study (Figure 3(d)). In huNOG mice, TRACP5b serum levels remained at a similar level during the study and were lower compared to NOG mice (Figure 3(d)). Staining of osteoclasts from tumor-bearing tibias was in line with the serum TRACP5b levels and showed a lower number of osteoclasts in stained sections obtained from huNOG mice (Figure 3(d)). The number of osteoblasts was increased in Donor 2 mice compared to NOG mice (Figure 3(f)). Taken together, huNOG mice have a lower number of osteoclasts and a higher number of osteoblasts in the tumor-bearing tibiae, resulting in higher tumor-induced formation of new bone.

As the study included immunodeficient NOG mice that have the same background as the huNOG mice, the effects of human immune cells on tumor growth in the bone can be assessed. The huNOG mice mainly support the development of human T and B cells, and we have shown that high numbers of these cells are present in the mice (Figure 1(c)), suggesting that these cells would be major contributors in the increased tumor growth in the bones of these mice. Generally, the role of T and B cells in bone remodeling is not well established [13]. When CD4+ cells were transferred to immunodeficient mice, they increased bone mass [27, 36], which could be explained by decreased osteoclast differentiation [36]. This could be further explained by the increased levels of OPG and RANKL both secreted by T cells [13]. Additionally, a high number of Tregs correlate with higher bone mass [13], and they can regulate osteoclastogenesis by secreting TGF*β*, IL-4, and IL-10 [36]. However, T cells can also trigger osteoblast maturation [13], and B cells can decrease the production of OPG which can lead to increased resorption and osteoporosis-like disease [31]. Therefore, both T and B cells are important regulators of homeostasis in the bone and also contribute to the formation of the osteoblastic bone reaction observed in this model.

### 3.4. Marker Expression and TILs in Tumors

**Figure 4:**
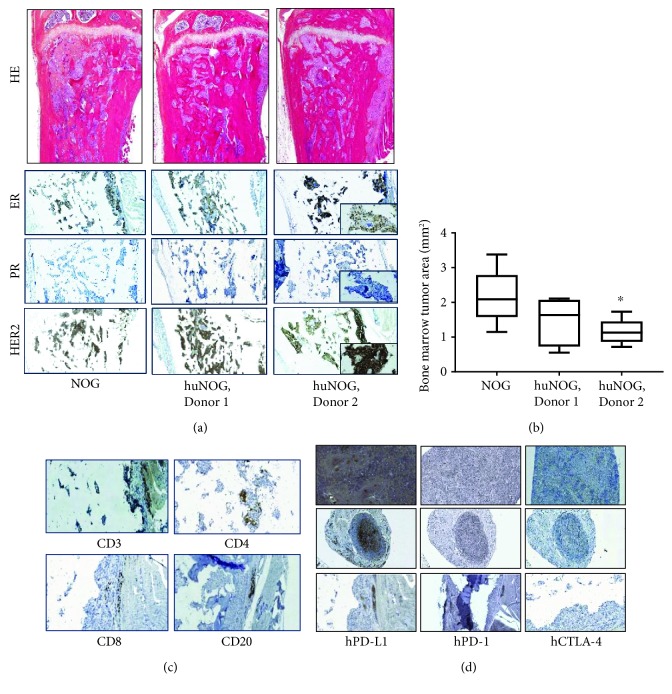
Tumor growth and TILs in NOG and huNOG mice. (a) Representative HE-stained sections of the tumor-bearing tibias of NOG and huNOG mice, magnification 5x. (b) Quantitation of the tumor area (only tumor cells) in the bone marrow (mm^2^, mean ± IQR25%±min/max). Statistical analysis was performed using ANOVA and pairwise comparisons using Dunnett's test. Statistical significance is marked as ^∗^ *p* < 0.05. (c) Representative images of CD3 (T cells), CD4 (T helper cells), CD8 (cytotoxic T cells), and CD20 (B cells) in tumors growing in the bone, magnification 20x. (d) Representative images of immune checkpoint inhibitors PD-L1 (programmed death-ligand 1), PD-1 (programmed cell death protein 1), and CTLA-4 (cytotoxic T lymphocyte-associated protein 4) in the spleen, lymph nodes, and tumors, magnification 20x.

HE staining of tumor-bearing tibia showed an increased bone mass area (Figure 4(a)) compared to the intact tibia on the same mice (Figure 2(a), Supplement 1). The tumor area in the bone marrow was quantitated from HE-stained sections, and it was smaller in huNOG mice engrafted with Donor 2 cells (Figure 4(b)), which is consistent with the increased bone area (Figures 3(a) and 3(b)). BT-474 cells expressed PR and HER2 but not ER in *in vitro* culture conditions (Supplement 3). When the tumors are growing in the bone marrow, the expression of ER was regained and PR expression was lost, and HER2 remained in the cells (Figure 4(a)). The ER, PR, and HER2 expressions were similar between NOG and huNOG mice (Figure 4(a)). The differences in marker expression between the *in vitro* and *in vivo* conditions may be due to differences in hormone amounts in these conditions.

The tumors were characterized for the TILs at the endpoint. In general, a low number of CD3-, CD4-, and CD8-positive T cells and CD20-positive B cells were observed (Figure 4(c)). These cells have been also observed in tumors growing in the bone by others [26]. The staining for these cells was low to negative in tumors, but a high number of these cells were observed in immune-related organs of huNOG mice (Figure 1(c)). Additionally, the expression of PD-L1 and PD-1 was analyzed and was low to negative in these tumors (Figure 4(d)). Also, PD-L1 was negative in BT-474 cells grown in the culture (Supplement 3). The expression of CTLA-4 was negative in all mice, also in immune-related organs (Figures 4(d) and 1(c)).

One of the issues unaddressed in this study was the early infiltration of T lymphocytes into the tumor. In this study, we only looked at the infiltration in late-stage bone metastatic tumors, a low number of T lymphocytes were observed, and the tumors were concluded to present “cold” or “immune desert” type. Typically at the early stage, the number of TILs is higher in the tumors and it would have been interesting to see if the immune cell infiltration would be higher at an earlier time point [8, 37]. This would have also been helpful in understanding what immune cells were contributing to increased tumor-induced bone changes observed in this model. However, HR+ breast cancer is typically TIL-low corresponding to what was observed in our model [8]. Furthermore, in patients, bone metastases are typically observed late and usually when they start to induce secondary effects such as fractures or bone pain [1, 2, 4, 5]. For this, a model resembling the condition of these cases is of great relevance. Additionally, the cold tumor type warrants for compounds or combinations of compounds to attract immune cells into the tumor, and these types of models are of high interest at the moment. For example, combinations with cytotoxic, hormonal, radiation, radiotherapy, and antiangiogenic compounds are carried out in preclinical and clinical studies [8, 10, 37–39].

## 4. Conclusions

In this study, we report the first establishment of a breast cancer bone growth model in humanized mice. BT-474 human breast cancer cells induced new bone growth in the model mimicking the formation of osteoblastic bone metastases in patients. The increased bone growth was associated with increased BMD in the mice. Additionally, the humanization process and/or the presence of human immune cells did not considerably affect the bone phenotype observed in the healthy bones of the mice. The BT-474 tumors present a cold tumor with a low number of immune cells. Some donor-related differences were observed, which should be taken into consideration when planning studies in humanized mice. Humanized mouse models provide an improved tool that can be used in preclinical efficacy evaluation of IO compounds also in the context of cancer bone metastasis.

## Data Availability

The data used to support the findings of this study are available from the corresponding author upon request.

## Supplementary Materials

Supplementary Materials Supplement 1: HE staining of the tumor-bearing and healthy tibia. Supplement 2: bone turnover markers in NOG and huNOG mice. Supplement 3: IHC stainings of BT-474 cells for ER, PR, HER2, and PD-L1.
