# Supplementary material for: Human Immune System Increases Breast Cancer-Induced Osteoblastic Bone Growth in a Humanized Mouse Model without Affecting Normal Bone
Source: J Immunol Res. 2019 May 9;2019:4260987. doi: 10.1155/2019/4260987 (PMC6532310; doi:10.1155/2019/4260987)
Supplement: Supplementary Materials — Supplement 1: HE staining of the tumor-bearing and healthy tibia. Supplement 2: bone turnover markers in NOG and huNOG mice. Supplement 3: IHC stainings of BT-474 cells for ER, PR, HER2, and PD-L1. [file 4260987.f1.pptx]

## Slide 1
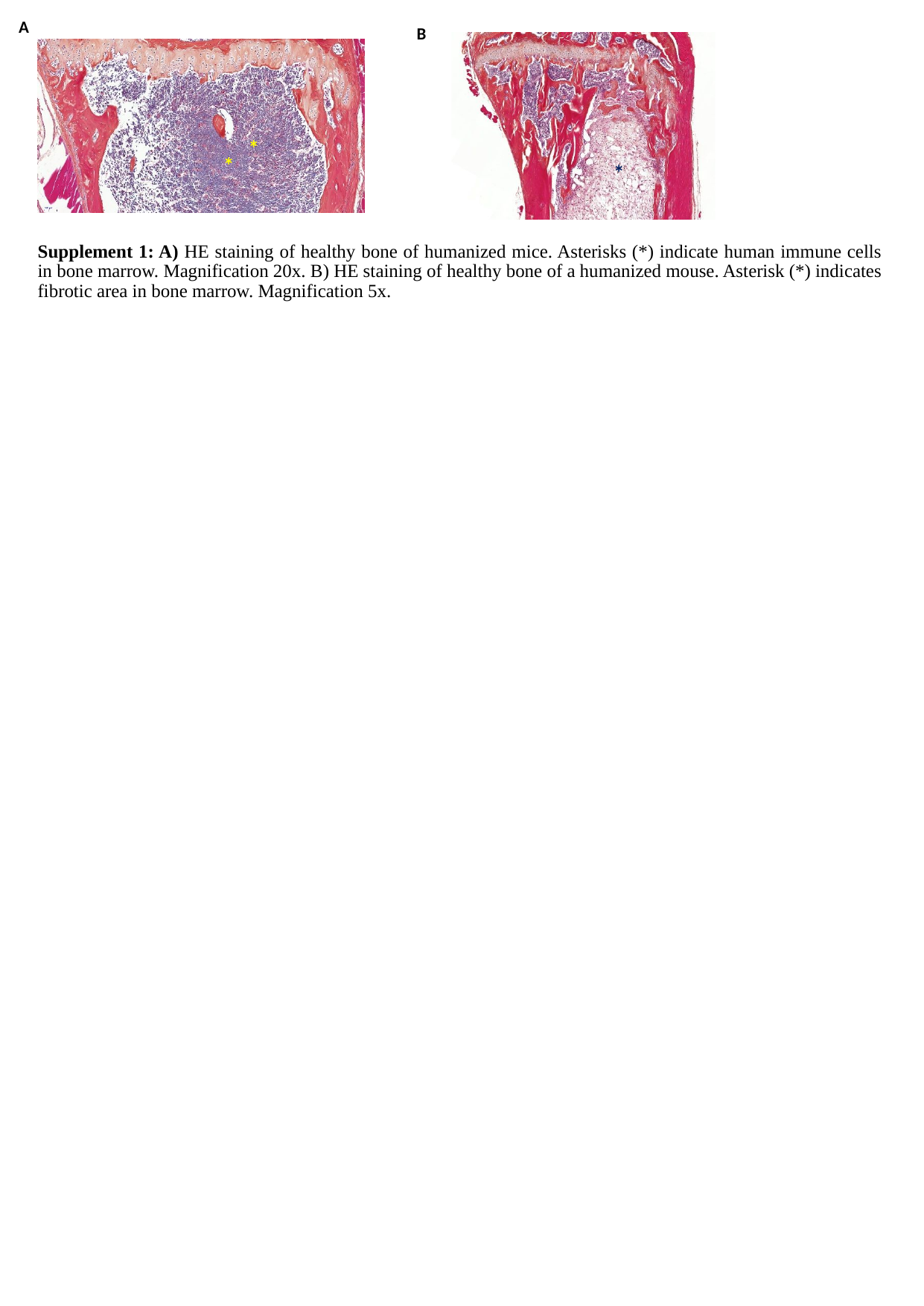

A
B
*
*
*
Supplement 1: A) HE staining of healthy bone of humanized mice. Asterisks (*) indicate human immune cells in bone marrow. Magnification 20x. B) HE staining of healthy bone of a humanized mouse. Asterisk (*) indicates fibrotic area in bone marrow. Magnification 5x.

## Slide 2
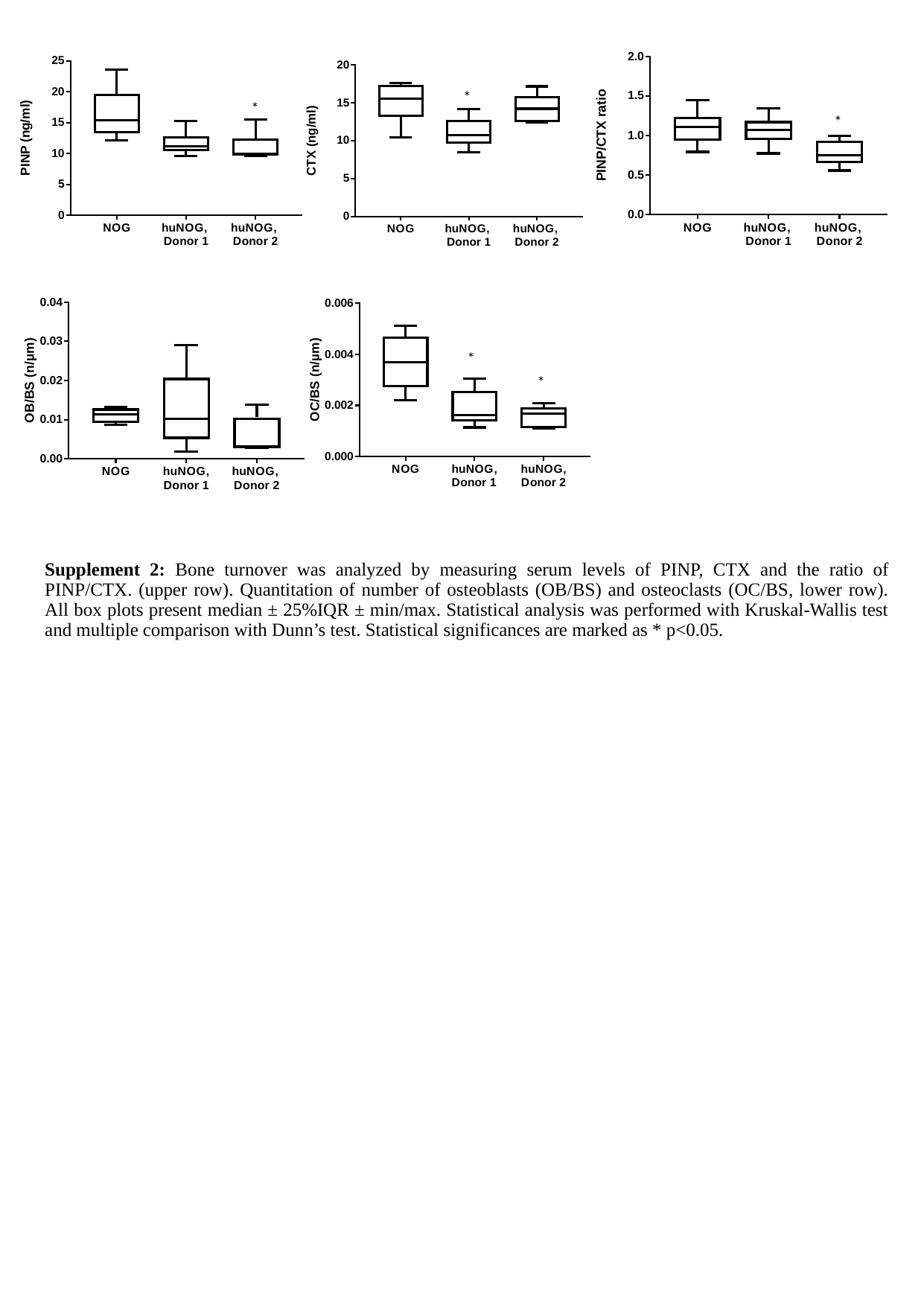

*
*
*
*
*
Supplement 2: Bone turnover was analyzed by measuring serum levels of PINP, CTX and the ratio of PINP/CTX. (upper row). Quantitation of number of osteoblasts (OB/BS) and osteoclasts (OC/BS, lower row). All box plots present median ± 25%IQR ± min/max. Statistical analysis was performed with Kruskal-Wallis test and multiple comparison with Dunn’s test. Statistical significances are marked as * p<0.05.

## Slide 3
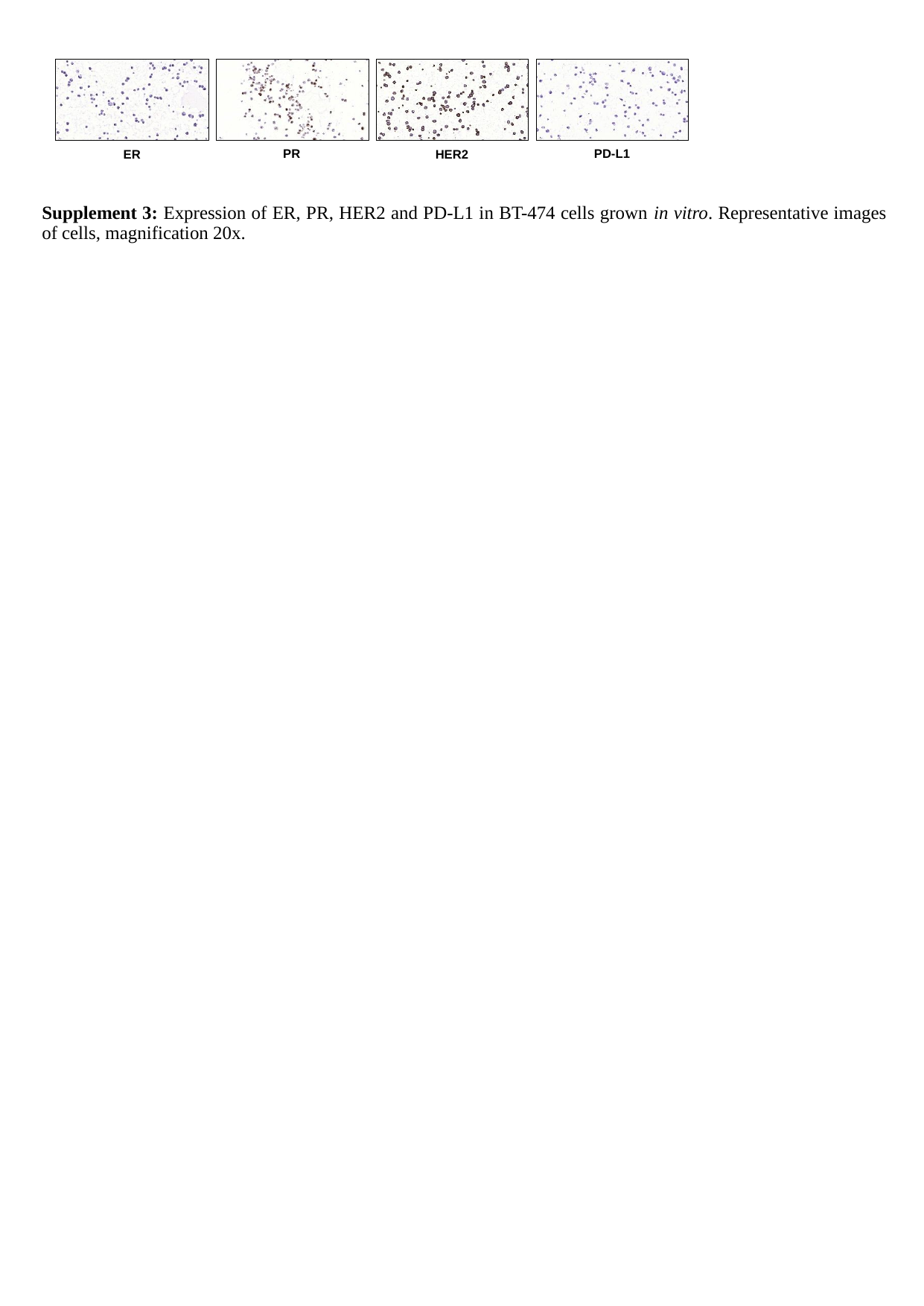

PR
PD-L1
HER2
ER
Supplement 3: Expression of ER, PR, HER2 and PD-L1 in BT-474 cells grown in vitro. Representative images of cells, magnification 20x.
